# Efficacy and safety analysis of induction therapy for unresectable locally advanced non-small-cell lung cancer: a retrospective study based on IPTW

**DOI:** 10.3389/fonc.2026.1902715

**Published:** 2026-09-15

**Authors:** Tianlong Chen, Hongxu Zhang, Hongtao Yin, Mingyan Li, Qian Zhang, Jiaao Liu, Hao Qi

**Affiliations:** 1 Department of Radiation Oncology, Harbin Medical University Cancer Hospital, Harbin, China; 2 Department of Breast Surgery, Harbin Medical University Cancer Hospital, Harbin, China

**Keywords:** chemoradiotherapy, immune checkpoint inhibitors, immune consolidation, immunotherapy, induction immunochemotherapy, locally advanced non–small cell lung cancer

## Abstract

**Background:**

Concurrent chemoradiotherapy (cCRT) followed by consolidation immunotherapy constitutes the current standard of care for unresectable locally advanced non–small cell lung cancer (LA-NSCLC). Despite this approach, a substantial proportion of patients experience disease progression, underscoring the need for novel therapeutic strategies. This study was undertaken to evaluate whether induction chemoimmunotherapy administered prior to definitive CRT can improve clinical outcomes. We evaluated the efficacy and safety of induction chemoimmunotherapy followed by CRT in patients with unresectable LA-NSCLC.

**Methods:**

A retrospective cohort of patients with unresectable LA-NSCLC treated at the Affiliated Cancer Hospital of Harbin Medical University between January 2020 and October 2022 was analyzed. Patients were classified according to receipt of induction immunochemotherapy into the I-CRT group (n=62) and the CRT group(n=142). Inverse probability of treatment weighting (IPTW) was applied to adjust for baseline confounding. Assess overall survival (OS), progression- free survival (PFS), and safety in the two patient groups.

**Results:**

After IPTW adjustment, the weighted cohort comprised 286 patients (I-CRT, 104; CRT, 182). In IPTW weighted analyses, median OS was significantly longer in the I-CRT group than in the CRT group (38.8 months vs 23.9 months; hazard ratio [HR], 0.46; 95% confidence interval [CI], 0.28 to 0.77; p = 0.003). Median PFS was also improved with induction chemoimmunotherapy (18.1 months vs 11.7 months; HR, 0.61; 95% CI, 0.39 to 0.95; p=0.028). The overall incidence of treatment-related adverse events (TRAE) was comparable between groups, with the exception of hyperthyroidism, which occurred more frequently in the I-CRT cohort (16. 1% vs 5.6%; p=0.025). Hematologic toxicity was the most common grade ≥3 TRAE. Collectively, the safety profile was acceptable and manageable.

**Conclusion:**

Induction chemoimmunotherapy administered prior to CRT confers significant and durable improvements in PFS and OS, with a manageable safety profile.

## Introduction

Lung cancer remains the most common malignancy worldwide and the leading cause of cancer-related mortality. Non–small cell lung cancer (NSCLC) accounts for approximately 85% of cases, and roughly one third of patients present with locally advanced (LA) disease, at which point the opportunity for curative resection is often lost (1, 2). The advent of immune checkpoint inhibitors has transformed the therapeutic landscape for LA-NSCLC, producing durable responses in a subset of patients (3, 4).

Notably, the phase III PACIFIC trial established consolidation durvalumab after platinum-based concurrent chemoradiotherapy (cCRT) as the standard of care for unresectable stage III disease; the most recent 5 year results reported a median overall survival of 47.5 months with durvalumab versus 29.1 months with placebo, confirming a substantial and sustained survival benefit (5).

Despite these advances, treatment failure remains common, with distant metastasis the predominant mode of progression (6). This pattern likely reflects, at least in part, occult metastatic disease present at diagnosis that escapes detection because of limitations in current staging practices (for example, variable use of whole-body PET/CT and endoscopic assessments) and barriers related to access and cost. Patients with unrecognized systemic disease are more appropriately treated with systemic therapy up front (7), since early emphasis on local radiotherapy may delay or reduce the intensity of systemic treatment and thereby permit distant progression. Accordingly, there is an urgent need to refine staging and patient selection and to investigate novel therapeutic strategies that better integrate systemic and local modalities to further improve outcomes in LA-NSCLC.

Although immunotherapy has markedly improved outcomes for patients with LA-NSCLC, a substantial proportion approaching half do not ultimately benefit from immune checkpoint inhibitors (ICIs), owing chiefly to disease progression after CRT or treatment-limiting toxicities that preclude ICI administration (8). Administering ICIs earlier, during the induction phase, could increase the likelihood that patients receive and derive benefit from immunotherapy (9). Supporting this strategy, the CheckMate-816 trial demonstrated that neoadjuvant chemoimmunotherapy significantly improved 5-year survival among patients with resectable stage IB–IIIA NSCLC (65.4% vs 55.0%; P = 0.048), establishing the clinical value of neoadjuvant chemoimmunotherapy in that population (10). Moreover, preliminary results from the prospective AFT- 16 study indicate that induction chemoimmunotherapy prior to CRT may yield survival benefit in unresectable LA-NSCLC (11). Together, these data provide a compelling rationale to further evaluate induction chemoimmunotherapy as a means to increase ICI exposure and improve outcomes in unresectable LA-NSCLC.

We conducted a retrospective cohort study of consecutive patients with unresectable LA-NSCLC treated at Harbin Medical University Cancer Hospital from January 2020 to October 2022. Clinical records were systematically reviewed to evaluate the efficacy and safety of induction immunochemotherapy administered prior to CRT.

To mitigate confounding and enhance the robustness of effect estimates, inverse probability of treatment weighting (IPTW) was used to balance baseline covariates in comparative analyses.

## Materials and methods

### Data sources and recruitment

We conducted a retrospective cohort study of patients with NSCLC who presented to the Harbin Medical University Cancer Hospital between January 2020 and October 2022. Eligible patients were aged ≥18 years, had histologic or cytologic confirmation of NSCLC, and were staged as clinical stage III according to the American Joint Committee on Cancer Staging Manual, 8th Edition Criteria. Patients receiving induction immunochemotherapy were required to have completed at least two treatment cycles. Exclusion criteria comprised prior radical resection for lung cancer; presence of sensitizing epidermal growth factor receptor (EGFR) mutations or Anaplastic lymphoma kinase (ALK) or ROS proto-oncogene 1 rearrangements; receipt of stereotactic body radiotherapy as the radiotherapy modality; and incomplete baseline, imaging, or follow-up data. Given the retrospective design, the requirement for written informed consent was waived (**Figure 1**).

**Figure 1:**
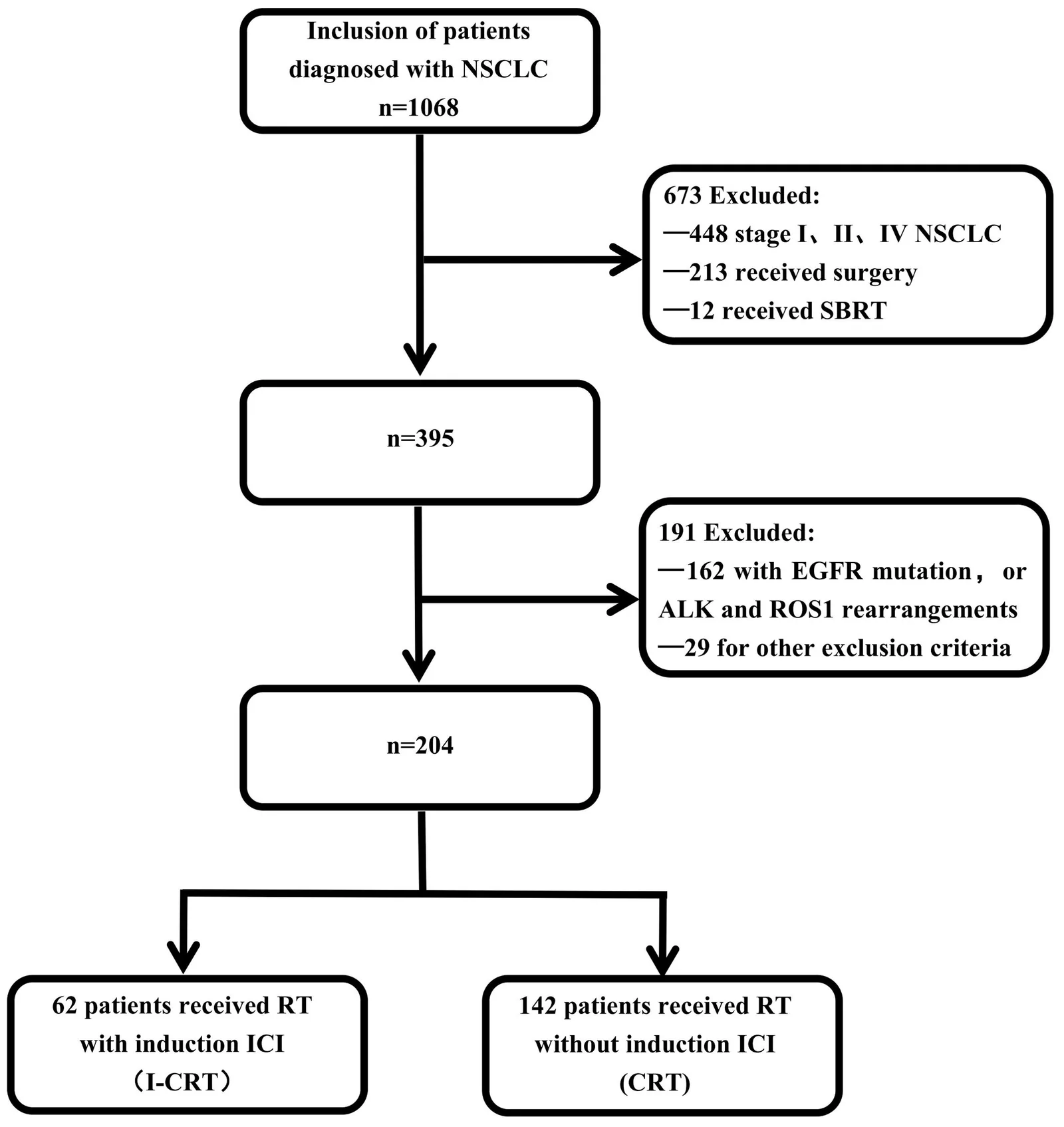
Patient inclusion flow chart. CRT, chemoradiotherapy; NSCLC, non-small cell lung cancer.

### Treatment

After screening, patients were allocated to two groups: radical chemoradiotherapy with or without immunotherapy consolidation (CRT group) and induction immunochemotherapy followed by radical chemoradiotherapy with or without immunotherapy consolidation (I- CRT group). Induction immunochemotherapy comprised 2–6 cycles. ICIs (tirelizumab, toripalimab, camrelizumab, and sintilimab) were used for induction, and all chemotherapy regimens were platinum-based doublets. All patients underwent CT- simulation–based radiotherapy planning.

Radiotherapy was delivered either concurrently with chemotherapy or sequentially, using intensity- modulated radiotherapy or volumetric- modulated arc therapy, with image- guided techniques employed during treatment to ensure positioning accuracy.

The prescribed dose was 60–66 Gy in 30–33 fractions to 95% of the planning target volume. Follow- up assessments were performed every 3 months during the first 2 years and every 6 months thereafter. Treatment and imaging data were collected from follow- up visits and institutional imaging reports for final analysis. The last follow- up date was 10 October 2025.

### Clinical outcomes and toxicity evaluation

Baseline demographic and treatment data were extracted from medical records, and all patients underwent protocol-specified routine follow-up. The primary endpoints were OS and PFS. OS was defined as the interval from treatment initiation to death from any cause. PFS was defined as the interval from treatment initiation to the first documented radiographic or clinical disease progression or death, assessed according to RECIST version 1.1. The key secondary endpoint was safety, with adverse events graded per the Common Terminology Criteria for Adverse Events (CTCAE) version 5.0.

### Statistical analysis

To mitigate confounding between treatment groups, IPTW was applied, a method validated as effective in multiple studies (12, 13). Propensity scores were estimated from a logistic regression model with treatment assignment (I-CRT versus CRT) as the dependent variable and nine prespecified baseline covariates: sex, age, smoking status, Eastern Cooperative Oncology Group Performance Status (ECOG-PS), pathological subtype, disease stage, radiotherapy dose, receipt of immunotherapy maintenance, and CRT sequencing. Covariate balance after weighting was evaluated using absolute standardized mean differences (SMD); an absolute SMD < 0.1 was predefined to indicate adequate balance. Time to event endpoints were summarized using IPTW weighted Kaplan–Meier estimates to obtain medians and landmark survival rates, and comparisons between groups were made using IPTW weighted log rank tests. Cox proportional hazards models incorporating IPTW were used to estimate hazard ratios (HR) and 95% confidence intervals (CI); univariate Cox models were used to evaluate the prognostic association of individual factors with survival outcomes, and exploratory subgroup analyses employed unstratified Cox models without adjustment for multiplicity. All statistical tests were two-sided, with P < 0.05 considered statistically significant. Analyses were performed in R (version 4.5.2).

## Result

### Patients

A total of 204 patients were included (I-CRT, n = 62; CRT, n = 142). Before IPTW adjustment, median age was 65 years (range 43–78) in the I-CRT group and 62 years (range 45–80) in the CRT group. The I-CRT cohort had a higher proportion of stage IIIC disease (29.0% vs 19.7%) and more frequent receipt of maintenance immunotherapy (38.7% vs 27.5%) than the CRT group. After IPTW weighting, absolute standardized mean differences for all prespecified covariates were <0.1, indicating adequate balance across baseline characteristics—including age, sex, smoking status, histologic subtype, and disease stage between the two groups (**Table 1**).

**Table 1:** Baseline characteristics and treatment information before and after IPTW.

Characteristics	Before IPTW				After IPTW			
	I-CRT N=62, (%)	CRT N=142, (%)	P	SMD	I-CRT* N=104, (%)	CRT* N=182, (%)	P	SMD
Sex			0.454	0.149			0.775	0.035
Male	54 (87. 1)	116 (81.7)			88 (84.3)	151 (83.0)		
Female	8 (12.9)	26 (18.3)			16 (15.7)	31 (17.0)		
Age			0.062	0.309			0.777	0.043
<65	29 (46.8)	88 (62.0)			57 (54.6)	103 (56.7)		
≥65	33 (53.2)	54 (38.0)			47 (45.4)	79 (43.3)		
Smoking			0.248	0.208			0.429	0.092
Yes	50 (80.6)	102 (71.8)			82 (78.5)	136 (74.6)		
No	12 (19.4)	40 (28.2)			22 (21.5)	46 (25.4)		
ECOG PS			0.162	0.217			0.963	0.013
0	24 (38.7)	41 (28.9)			34 (32.7)	58 (31.9)		
1	35 (56.5)	91 (64. 1)			63 (60.6)	112 (61.5)		
2	3 (4.8)	10 (7.0)			7 (6.7)	12 (6.6)		
History			0.616	0.153			0.961	0.025
Squamous	49 (79.0)	104 (73.2)			79 (76.0)	136 (74.9)		
Adenocarcinoma	11 (17.7)	34 (23.9)			22 (21.2)	40 (21.8)		
Others	2 (3.2)	4 (2.8)			3 (2.9)	6 (3.3)		
Stage			0.342	0.218			0.834	0.067
IIIA	20 (32.3)	52 (36.6)			38 (36.2)	65 (35.7)		
IIIB	24 (38.7)	62 (43.7)			40 (38.9)	76 (41.8)		
IIIC	18 (29.0)	28 (19.7)			26 (24.9)	41 (22.5)		
Radiation dose			0.292	0.184			0.770	0.036
<60Gy	19 (30.6)	32 (22.5)			23 (21.7)	43 (23.9)		
≥60Gy	43 (69.4)	110 (77.5)			81 (78.3)	139 (76.1)		
Consolidation ICIS			0.152	0.241			0.634	0.058
Yes	24 (38.7)	39 (27.5)			36 (34.2)	58 (32.1)		
No	38 (61.3)	103 (72.5)			68 (65.8)	124 (67.9)		
Sequence CRT			0.270	0.199			0.922	0.012
Sequential	49 (79.0)	100 (70.4)			76 (73.2)	134 (73.6)		
Concurrent	13 (21.0)	42 (29.6)			28 (26.8)	48 (26.4)		

*Following IPTW, the effective sample size differed from the original cohort due to weighting. The actual number of patients, however, remained unchanged. As the P-value was not close to the significance threshold, percentages were rounded to whole numbers for presentation.

### Treatment

All patients in the I-CRT arm received induction immunochemotherapy; 43 of 62 patients (69.4%) completed ≥4 cycles, including 16 (25.8%) who received 4 cycles and 18 (29.0%) who received 6 cycles. A greater proportion of patients in the CRT arm completed a radiotherapy dose ≥60 Gy (77.5% vs 69.5%), whereas immunoconsolidation was more frequent in the I-CRT arm (38.7% vs 27.5%). The objective response rate (ORR) to induction immunochemotherapy was 61.3% (38 of 62).A total of 24 patients in the I-CRT group and 39 patients in the CRT group completed consolidation immunotherapy (**Table 2**). By number of induction cycles, ORR was 52. 1% among patients receiving 2–4 cycles versus 65.4% among those receiving ≥5 cycles; this difference was not statistically significant (p = 0.318).

**Table\ 2:** Summary of treatment and tumor response.

Characteristics	Overall N=204, (%)	I-CRT N=62, (%)	CRT N=142, (%)
Cycles of induction ICI			
\ 2	5 (2.5)	5 (8. 1)	
\ 3	14 (6.9)	14 (22.6)	
\ 4	16 (7.8)	16 (25.8)	
\ 5	9 (4.4)	9 (14.5)	
\ 6	18 (8.8)	18 (29.0)	
Best response to induction immunotherapy			
\ CR	0 (0.0)	0 (0.0)	
\ PR	38 (18.6)	38 (61.3)	
\ SD	23 (11.3)	23 (37. 1)	
\ PD	1 (0.5)	1 (1.6)	
Best response to CRT			
\ CR	4 (2.0)	2 (3.2)	2 (1.4)
\ PR	140 (68.6)	47 (75.8)	93 (65.5)
\ SD	55 (27.0)	11 (17.7)	44 (31.0)
\ PD	5 (2.5)	2 (3.2)	3 (2. 1)
PD-L1 expression			
\ \<1%	22 (10.8)	7 (11.3)	15 (10.6)
\ 1-49%	27 (13.2)	8 (12.9)	19 (13.4)
\ \≥50%	22 (10.8)	10 (16. 1)	12 (8.5)
\ Missing	133 (65.2)	37 (59.7)	96 (67.6)
Duration of consolidation ICI			
\ None	141 (69.1)	38 (61.3)	103 (72.5)
\ \<12 months	34 (16.7)	14 (22.6)	20 (14.1)
\ \≥12 months	29 (14.2)	10(16. 1)	19 (13.4)

### Survival outcomes before IPTW

In the unweighted overall population, median follow-up was 21.5 months (IQR 16.1–29. 1). At the data cutoff (October 10, 2025), 117 deaths had occurred: 30 of 62 (48.4%) in the I-CRT arm and 87 of 142 (61.3%) in the CRT arm. Compared with CRT, I-CRT was associated with a statistically significant reduction in the risk of death (HR 0.60; 95% CI, 0.39–0.91; p = 0.015). Median OS was 34.7 months (95% CI, 27.7–41.7) in the I-CRT group versus 24.5 months (95% CI, 21.6–28.0) in the CRT group; corresponding 1-, 3-, and 5-year OS rates were 88.7%, 42.2%, and 33.6% for I-CRT and 83.8%, 26.5%, and 12.3% for CRT, respectively.

For PFS, the I-CRT arm showed a numerically favorable but not statistically significant improvement versus CRT (HR 0.72; 95% CI, 0.51–1.01; p = 0.055). Median PFS was 16.3 months (95% CI, 12.3–23.4) with I-CRT versus 11.7 months (95% CI, 9.6–15.8) with CRT; 1-, 2-, and 3-year PFS rates were 62.9%, 33.7%, and 22.5% for I-CRT and 49.3%, 24.3%, and 13.0% for CRT, respectively (**Figures 2A, B**).

**Figure 2:**
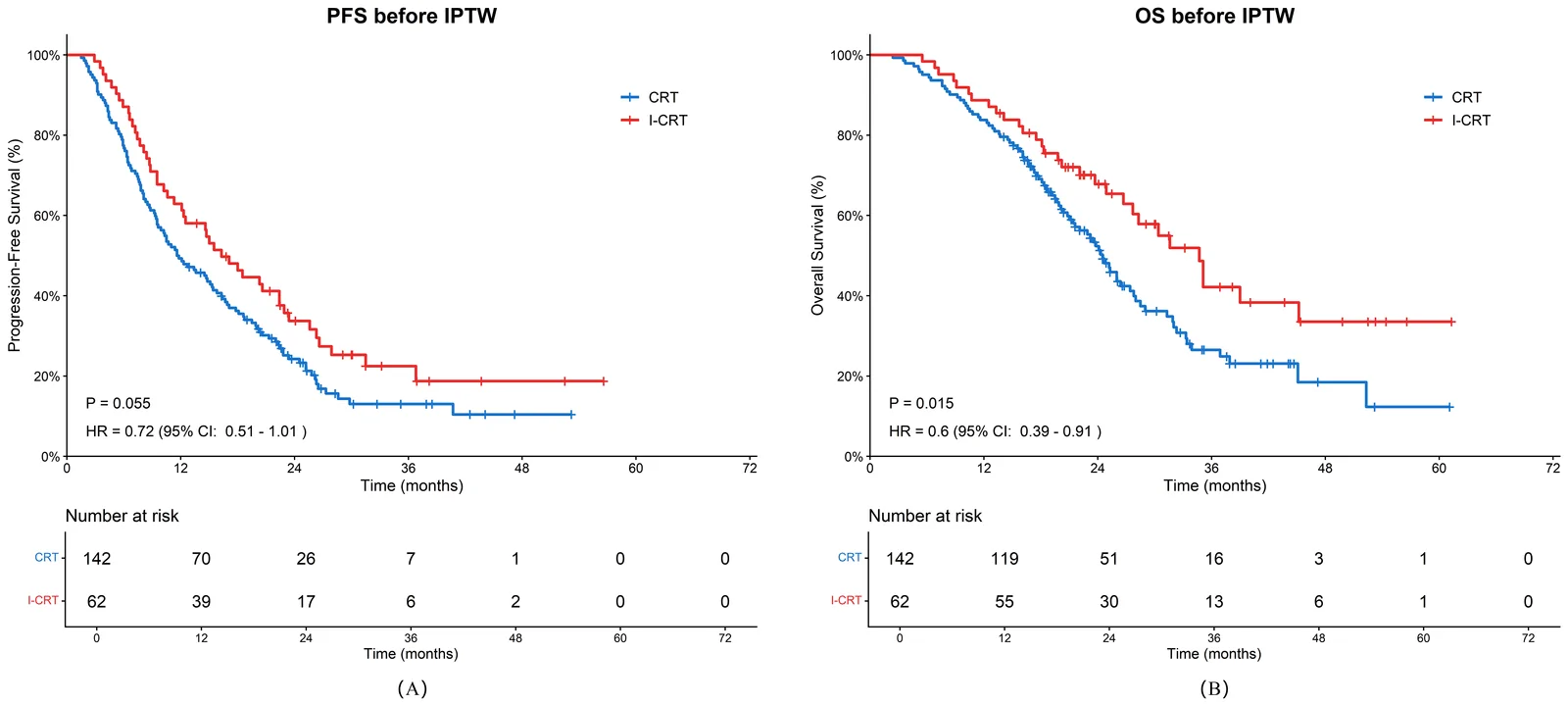
**(A)** Overall survival (OS) of the I-CRT group and the CRT group before IPTW. **(B)** Progression-free survival (PFS) of the I-CRT group and the CRT group before IPTW.

Supplementary Cox regression analyses restricted to patients in the I-CRT group were performed to identify prognostic factors associated with survival outcomes. In the multivariable analysis, receipt of consolidation ICIs remained independently associated with improved PFS (HR, 0.48; 95% CI, 0.24–0.96; P = 0.038) and OS (HR, 0.58; 95% CI, 0.36–0.94; P = 0.026). In addition, earlier disease stage and a radiation dose ≥60 Gy were independently associated with improved OS (**Supplementary Table 1**).

Univariable and multivariable Cox proportional hazards regression analyses were conducted in the unweighted cohort to evaluate factors associated with PFS and OS (**Supplementary Table 2**). After adjustment for potential confounders, I-CRT remained an independent predictor of improved PFS (HR, 0.63; 95% CI, 0.44–0.91; P = 0.014) and OS (HR, 0.60; 95% CI, 0.39–0.91; P = 0.015). A radiation dose of ≥60 Gy was independently associated with better PFS, while consolidation ICI therapy was independently associated with improved OS. In contrast, sequential CRT was associated with inferior PFS and OS.

### Survival outcomes after IPTW

After IPTW weighting, the survival benefit with I- CRT remained robust. I- CRT was associated with a significant reduction in the risk of death compared with CRT (HR 0.46; 95% CI, 0.28–0.77; p = 0.003), corresponding to a 54% relative risk reduction.

Median OS was 38.8 months (95% CI, 32.4–45.2) with I- CRT versus 23.9 months (95% CI, 21.5–27. 1) with CRT; the estimated 1-, 3-, and 5- year OS rates were 90.6%, 52.4%, and 43.6% for I- CRT versus 80.5%, 30.3%, and 14.5% for CRT, respectively.

Following IPTW adjustment, I- CRT also demonstrated a statistically significant improvement in PFS (HR 0.61; 95% CI, 0.39–0.95; p = 0.028). Median PFS was 18.1 months (95% CI, 14.6–24.9) with I- CRT versus 11.7 months (95% CI, 9.9–15.2) with CRT; corresponding 1-, 2-, and 3- year PFS estimates were 61.4%, 40.9%, and 29.5% for I- CRT and 49.3%, 25.8%, and 12.7% for CRT(**Figures 3A**, **B**).

**Figure 3:**
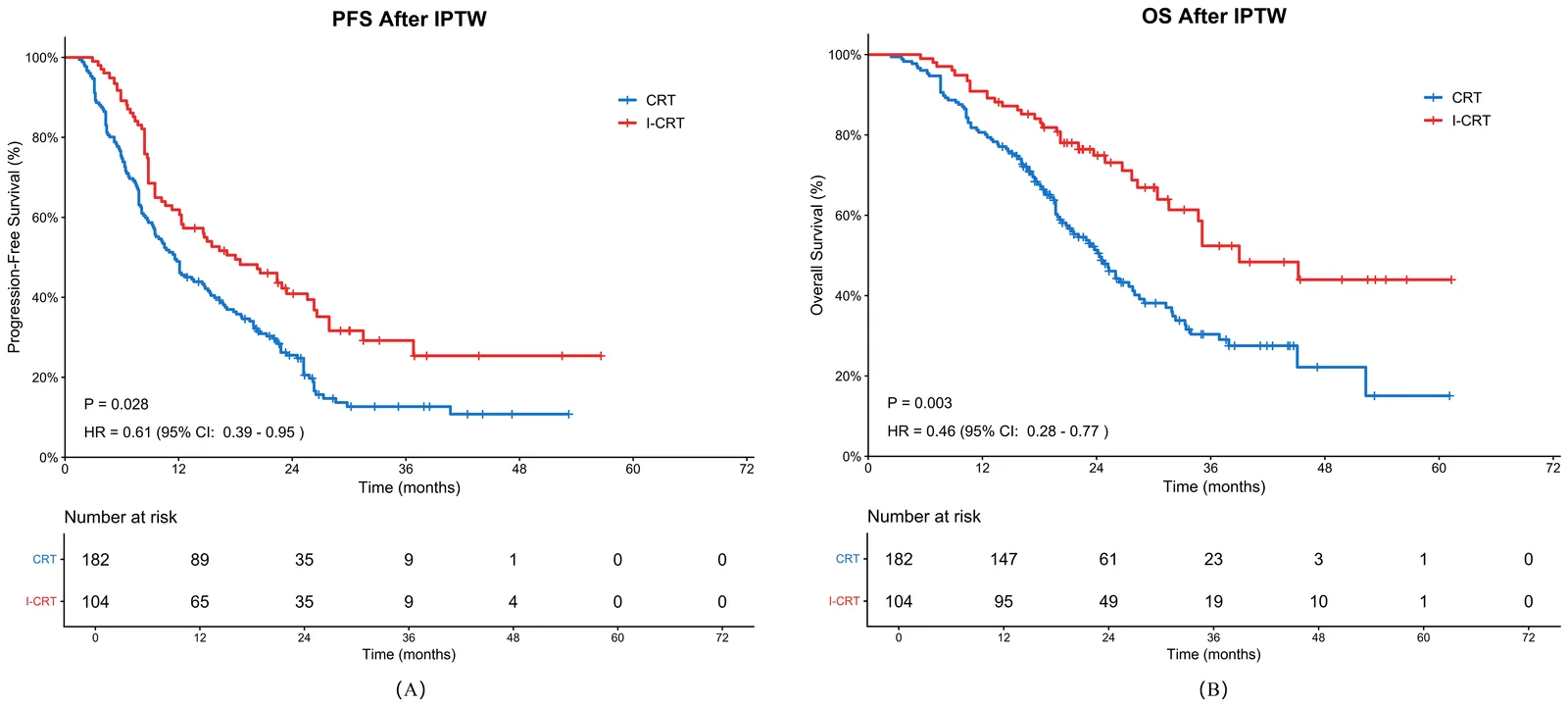
**(A)** Overall survival (OS) of the I-CRT group and the CRT group after IPTW. **(B)** Progression-free survival (PFS) of the I-CRT group and the CRT group after IPTW.

### Safety

Before IPTW adjustment, the overall safety profile was comparable between groups. Treatment-related adverse events (TRAE) of any grade occurred in 93.5% of patients in the I-CRT arm and 90.8% in the CRT arm. The incidence of radiation pneumonitis by grade in the I-CRT versus CRT arms was 34.0% versus 28.5% for grade 1–2, 4.0% versus 3.4% for grade 3–4, and 2.0% versus 1.4% for grade 5, with no statistically significant differences observed. One patient in the I-CRT cohort died of grade 5 pneumonia during CRT after completing induction therapy; this event was possibly immune-related. No other treatment-related deaths were recorded.

The only toxicity that was significantly more frequent in the I-CRT arm was hyperthyroidism, which was uniformly grade 1–2 and clinically mild. Otherwise, adverse event rates did not differ meaningfully between groups. In the I-CRT arm, the most common grade 1–2 events were leukopenia (53.2%), elevated creatinine (33.9%), and pneumonia (29.0%). Hematologic toxicities were the predominant ≥grade 3 events, principally leukopenia (9.7%) and thrombocytopenia (8. 1%). Overall, induction immunochemotherapy prior to CRT did not result in a clinically meaningful increase in severe adverse events; toxicity was manageable and the safety profile was similar to that of CRT alone (**Table 3**).

**Table 3:** Comparison of toxicities between groups before IPTW adjustment.

Toxicities	I-CRT (n=62)				CRT (n=142)				P
	All grade	Grade 1–2	Grade 3–4	Grade 5	All grade	Grade 1–2	Grade 3–4	Grade 5	
Any event	58 (93.5)	46 (74.2)	11 (17.7)	1 (1.6)	129 (90.8)	109 (76.8)	18 (12.7)	2 (1.4)	0.537
Pneumonitis	24 (38.7)	18 (29.0)	5 (8. 1)	1 (1.6)	48 (33.8)	39 (27.5)	7 (4.9)	2 (1.4)	0.513
Hypothyroidism	7 (11.3)	7 (11.3)	0 (0.0)	0 (0.0)	12 (8.5)	11 (7.7)	1 (0.7)	0 (0.0)	0.195
Hyperthyroidism	10 (16. 1)	10 (16. 1)	0 (0.0)	0 (0.0)	8 (5.6)	8 (5.6)	0 (0.0)	0 (0.0)	0.025*
Leukopenia	39 (62.9)	33 (53.2)	6 (9.7)	0 (0.0)	91 (64. 1)	80 (56.3)	11 (7.7)	0 (0.0)	0.874
Anemia	16 (25.8)	15 (24.2)	1 (1.6)	0 (0.0)	25 (17.6)	23 (16.2)	2 (1.4)	0 (0.0)	0.180
Thrombocytopenia	19 (30.6)	14 (22.6)	5 (8. 1)	0 (0.0)	38 (26.8)	29 (20.4)	9 (6.3)	0 (0.0)	0.577
Elevated creatinine	23 (37. 1)	21 (33.9)	2 (3.2)	0 (0.0)	48 (33.8)	44 (31.0)	4 (2.8)	0 (0.0)	0.653
Transaminase									
elevation	8 (12.8)	6 (9.7)	2 (3.2)	0 (0.0)	11 (7.7)	8 (5.6)	3 (2. 1)	0 (0.0)	0.259

*P < 0.05.

### Sensitivity analysis

To further address the potential bias introduced by differences in subsequent immunotherapy exposure, we performed a sensitivity analysis restricted to patients who received consolidation immunotherapy. Patients receiving consolidation immunotherapy in the I-CRT and CRT cohorts were defined as the I-CRT-I and CRT-I groups, respectively. After IPTW adjustment, all baseline covariates were well balanced between the two groups, with standardized mean differences (SMDs) below 0.1 (**Supplementary Table 3**), indicating adequate comparability for subsequent survival analyses.

In the unweighted cohort, the I-CRT-I group demonstrated significantly improved OS and PFS compared with the CRT-I group (OS, P = 0.011; PFS, P = 0.047) (**Supplementary Figure 1**). After IPTW adjustment, the survival advantage of I-CRT-I remained significant, with superior OS (P = 0.018) and PFS (P = 0.040) relative to the CRT-I group (**Supplementary Figure 2**). These findings further support the robustness of the primary analysis.

## Discussion

Our findings indicate that induction immunochemotherapy administered prior to CRT is feasible, generally well tolerated, and may provide a clinically meaningful survival benefit in patients with unresectable LA-NSCLC. Historically, approaches that added conventional cytotoxic chemotherapy, either as induction chemotherapy before CRT or as consolidation chemotherapy after CRT, did not improve survival in this population (14, 15). The advent of immune checkpoint inhibitors has reshaped the treatment paradigm: the PACIFIC regimen established consolidation immunotherapy after CRT as the standard of care, yet in routine practice a substantial proportion of patients fail to receive consolidation because of rapid disease progression or treatment- limiting toxicities following CRT (8).

Optimizing the PACIFIC framework by moving immunotherapy earlier in the treatment sequence is therefore an active area of investigation. Consistent with this rationale, the KEYNOTE-799 program evaluated concurrent chemo-immunoradiotherapy followed by consolidation immunotherapy and reported objective response rates exceeding 70% across cohorts, with encouraging PFS and OS signals; nevertheless, grade ≥3 treatment-related adverse events approached 65%, underscoring a nontrivial toxicity burden with that approach (16). The phase III PACIFIC- 2 trial evaluated whether adding immunotherapy concurrently to cCRT followed by immunotherapy consolidation improved outcomes versus cCRT followed by the same immunotherapy consolidation. Although PACIFIC- 2 showed numerical improvements in PFS and OS with the concurrent immunotherapy strategy, these differences did not reach statistical significance, and the concurrent approach resulted in increased treatment related toxicity (17). Similarly, the phase III CheckMate- 73L study found no survival benefit from combining immunotherapy with CRT in unresectable LA- NSCLC, and reported grade ≥3 treatment- related adverse events in approximately half of patients (18). Accordingly, concurrent administration of immunotherapy with CRT should be approached with caution until more definitive evidence is available.

An alternative strategy is to move immunotherapy earlier in the course of treatment as induction immunochemotherapy followed by sequential CRT. This approach may broaden access to immunotherapy for more patients, exploit potential synergy between chemotherapy and immune modulation, and enable tumor shrinkage during the induction phase that can reduce radiotherapy target volumes and thereby lower radiation related toxicity (19). Supporting evidence includes the prospective AFT- 16 study (n=62), in which patients received 2–4 cycles of atezolizumab induction followed by cCRT and subsequent immune maintenance (with immediate cCRT if progression occurred after two induction cycles) (11). Median OS was not reached, median PFS was 30.0 months (95% CI, 15.8–not reached), 12- and 24-month OS rates were 87.0% and 73.7%, and 12- and 24-month PFS rates were 68.9% and 54.2%, indicating promising efficacy. The multicenter single- arm phase II APOPO trial of three cycles of induction immunochemotherapy followed by cCRT and immune maintenance reported a median follow- up of 29.6 months and 12- month PFS and OS rates of 68.4% and 86.8%, respectively (20). In our cohort, the I- CRT group achieved 1-, 3-, and 5- year OS rates of 88.7%, 42.2%, and 33.6%, respectively, and 1-, 2-, and 3- year PFS rates of 62.9%, 33.7%, and 22.5%, respectively. These outcomes are comparable to the prospective trials at 1 year but diverge thereafter with lower survival beyond 2 years. A likely contributor is the relatively low rate of immune consolidation in our series (38.7%), underscoring the importance of optimizing adherence to consolidation immunotherapy. Collectively, the prospective trials and our data suggest that induction immunochemotherapy followed by sequential CRT is a viable and promising strategy for unresectable LA- NSCLC, but definitive conclusions require large- scale randomized controlled trials to confirm long term efficacy and safety.

LA-NSCLC patients frequently present with a large tumor burden, and induction immunochemotherapy can meaningfully reduce tumor volume during the induction phase, thereby decreasing the radiation target and facilitating subsequent definitive CRT (10, 21). In our cohort the I-CRT group had a higher proportion of advanced disease (stage IIIC, 29.0%), and in many cases immediate radiotherapy would have resulted in an unacceptably large lung volume receiving ≥20 Gy, making standard CRT difficult to deliver. Thus, induction immunochemotherapy to debulk tumor prior to CRT represents a rational strategy in this setting.

In our study most patients in the I-CRT cohort received ≥4 cycles of induction therapy; however, objective response rates did not differ significantly between patients who received 2–4 cycles and those who received ≥5 cycles. Published data are inconsistent regarding the optimal number of induction cycles: Deng et al. (21) reported that 3–4 cycles may be superior to 2 cycles, whereas Wu et al. (22) suggested 2–3 cycles could be preferable to ≥4 cycles. Taken together, the optimal duration of induction immunochemotherapy remains undetermined and should be individualized (2–4 cycles is a pragmatic choice in many cases), because prolonged induction may delay initiation of CRT and increase resource use. Given the retrospective design and limited sample size of our study, definitive conclusions cannot be drawn; prospective, ideally randomized, studies are warranted to establish the optimal number of induction cycles and the best sequencing of immunochemotherapy with definitive CRT.

Our safety analysis showed no statistically significant difference in the incidence of grade ≥3 TRAE between the I-CRT and CRT cohorts: 11 patients (17.7%) versus 18 patients (12.7%), respectively. The I-CRT group experienced an all-grade pneumonia rate of 38.7% and a grade ≥3 pneumonia rate of 8. 1%. For comparison, the PACIFIC trial reported all-grade and grade ≥3 pneumonia rates of 33.9% and 4.2%, respectively5. Differences in baseline characteristics and trial entry criteria are likely contributors to these discordant rates; notably, PACIFIC excluded patients with grade ≥2 pneumonitis following prior CRT, thereby removing a population at higher risk for pulmonary toxicity. Real world data are informative in this regard. In a cohort of 1,994 patients with LA-NSCLC, all-grade pneumonia occurred in 11.0% versus 6.8% p=0.0005 and grade ≥3 pneumonia in 8.4% versus 6.0% p=0.007 for ICI-treated versus CRT-only patients, respectively, a pattern broadly consistent with our observations. Intriguingly, subgroup analysis in that series showed a ~50% lower risk of grade ≥2 pneumonia among smokers compared with never smokers (p<0.001) (23), underscoring the potential influence of patient characteristics on pulmonary toxicity risk. Given concerns about cumulative toxicity and to improve tolerability, investigators have explored de-chemotherapy approaches. The SPRINT trial, which omitted concurrent chemotherapy and used radiotherapy followed by immune consolidation after three cycles of induction immunotherapy, reported promising early outcomes (1-year OS 92% and PFS 76%) with a manageable safety profile; the most common grade 3 adverse events were colitis and esophagitis (8% each), and no higher-grade toxicities were observed (24). Modifications in radiotherapy technique, including hypofractionation and proton therapy, have also shown early potential to reduce treatment-related toxicity (25, 26). In summary, induction immunochemotherapy may be associated with a higher incidence of severe adverse events in some series, but overall toxicities are generally controllable with appropriate patient selection and management. Cross-study differences in toxicity rates should be interpreted in the context of enrollment criteria, baseline patient characteristics, and treatment sequencing. Prospective studies are needed to better define optimal treatment paradigms that maximize efficacy while minimizing clinically meaningful toxicity.

This study has several limitations. First, the ICIs administered were heterogeneous across the cohort, which may have influenced both efficacy and safety estimates. Second, the retrospective observational design limits causal inference: although inverse probability of treatment weighting was used to mitigate measured confounding, residual confounding and unmeasured bias cannot be excluded, and prospective randomized trials are needed to validate these results. Third, programmed death-ligand 1 (PD-L1) status was missing for a large proportion of patients, precluding reliable PD- L1 stratified efficacy analyses. Fourth, exclusion of patients with incomplete baseline or treatment data may have introduced selection bias and further confounding, potentially affecting the precision and generalizability of our estimates.

## Conclusion

In conclusion, for patients with unresectable LA-NSCLC, induction immunochemotherapy followed by definitive CRT and subsequent immune consolidation is a feasible treatment strategy that may improve survival outcomes.

Prospective, adequately powered randomized controlled trials are required to generate definitive, high-level evidence to support the clinical implementation of this treatment strategy.

## Data Availability

The raw data supporting the conclusions of this article will be made available by the authors, without undue reservation.
