# Supplementary material for: Efficacy and safety analysis of induction therapy for unresectable locally advanced non-small-cell lung cancer: a retrospective study based on IPTW
Source: Front Oncol. 2026 Sep 15;16:1902715. doi: 10.3389/fonc.2026.1902715 (PMC13619472; doi:10.3389/fonc.2026.1902715)
Supplement: Supplementary file 1 [file Table1.docx]

Supplementary Table S1 Univariate and multivariate analyses of survival outcomes (I‑CRT group, before IPTW.

| **Characteristics** | **PFS** | | | | **OS** | | | |
| --- | --- | --- | --- | --- | --- | --- | --- | --- |
|  | Univariate analysis | | Multivariable analysis | | Univariate analysis | | Multivariable analysis | |
|  | HR (95% CI) | p value | HR (95% CI) | p value | HR (95% CI) | p value | HR (95% CI) | p value |
| **Age** |  |  |  |  |  |  |  |  |
| <65 vs ≥65 | 1.03 (0.57,1.84) | 0.931 |  |  | 1.09 (0.52, 2.27) | 0.814 |  |  |
| **Sex** |  |  |  |  |  |  |  |  |
| Male vs Female | 0.45 (0.16, 1.26) | 0.128 |  |  | 0.59 (0.18, 1.94) | 0.384 |  |  |
| **Smoking** |  |  |  |  |  |  |  |  |
| Yes vs No | 1.51 (0.82, 2.77) | 0.185 |  |  | 1.26 (0.72, 2.22) | 0.406 |  |  |
| **ECOG PS** |  |  |  |  |  |  |  |  |
| 0 vs 1-2 | 0.97 (0.40, 2.40) | 0.954 |  |  | 0.71 (0.33, 1.52) | 0.375 |  |  |
| **History** |  |  |  |  |  |  |  |  |
| Squamous vs Non-Squamous | 0.74 (0.49, 1.23) | 0.375 |  |  | 0.97 (0.40, 2.40) | 0.954 |  |  |
| **Stage** |  |  |  |  |  |  |  |  |
| IIIA vs IIIB and IIIC | 0.86 (0.49, 1.51) | 0.591 |  |  | 0.43(0.21, 0.92) | 0.025* | 0.45 (0.19, 0.99) | 0.048* |
| **Radiation dose** |  |  |  |  |  |  |  |  |
| ≥60Gy vs <60Gy | 0.68 (0.45, 1.06) | 0.085 |  |  | 0.46 (0.28, 0.76) | 0.011* | 0.57 (0.34, 0.97) | 0.041* |
| **Consolidation ICIS** |  |  |  |  |  |  |  |  |
| Yes vs No | 0.44(0.21, 0.92) | 0.029* | 0.48(0.24,0.96) | 0.038* | 0.51 (0.31, 0.83) | 0.007** | 0.58 (0.36, 0.94) | 0.026* |
| **Sequence CRT** |  |  |  |  |  |  |  |  |
| Sequential vs Concurrent | 1.52 (0.98,2.31) | 0.057 |  |  | 1.37(0.83, 2.26) | 0.218 |  |  |
| **Induction cycles** |  |  |  |  |  |  |  |  |
| 2-4 vs 5-6 | 0.72 (0.39, 1.33) | 0.297 |  |  | 0.67 (0.34, 1.49) | 0.359 |  |  |

Supplementary Table S2 Univariable and Multivariable Analyses of Survival Outcomes in the Unweighted Cohort.

| **Characteristics** | **PFS** | | | | **OS** | | | |
| --- | --- | --- | --- | --- | --- | --- | --- | --- |
|  | Univariate analysis | | Multivariable analysis | | Univariate analysis | | Multivariable analysis | |
|  | HR (95% CI) | p value | HR (95% CI) | p value | HR (95% CI) | p value | HR (95% CI) | p value |
| **Treatment group** |  |  |  |  |  |  |  |  |
| I-CRT vs CRT | 0.72 (0.51, 0.93) | 0.036* | 0.63 (0.44, 0.91) | 0.014* | 0.67 (0.46, 0.98) | 0.042* | 0.60 (0.39, 0.91) | 0.015* |
| **Age** |  |  |  |  |  |  |  |  |
| <65 vs ≥65 | 0.82 (0.56, 1.20) | 0.251 |  |  | 0.78 (0.46, 1.30) | 0.285 |  |  |
| **Sex** |  |  |  |  |  |  |  |  |
| Male vs Female | 0.89 (0.45, 1.77) | 0.732 |  |  | 0.82 (0.35, 1.91) | 0.645 |  |  |
| **Smoking** |  |  |  |  |  |  |  |  |
| Yes vs No | 1.15 (0.80, 1.65) | 0.439 |  |  | 1.11 (0.73, 1.70) | 0.622 |  |  |
| **ECOG PS** |  |  |  |  |  |  |  |  |
| 0 vs 1-2 | 0.80 (0.56, 1.22) | 0.312 |  |  | 0.64 (0.37, 1.12) | 0.144 |  |  |
| **History** |  |  |  |  |  |  |  |  |
| Squamous vs Non-Squamous | 1.14 (0.80, 1.62) | 0.470 |  |  | 0.93 (0.63, 1.39) | 0.738 |  |  |
| **Stage** |  |  |  |  |  |  |  |  |
| IIIA vs IIIB and IIIC | 0.82 (0.59, 1.13) | 0.222 |  |  | 0.67 (0.45, 1.01) | 0.052 |  |  |
| **Radiation dose** |  |  |  |  |  |  |  |  |
| ≥60Gy vs <60Gy | 0.70 (0.51, 0.96) | 0.029* | 0.61 (0.40, 0.93) | 0.022* | 0.76 (0.54, 1.07) | 0.110 |  |  |
| **Consolidation ICIS** |  |  |  |  |  |  |  |  |
| Yes vs No | 0.64 (0.46, 0.90) | 0.011* | 0.72 (0.51, 1.03) | 0.076 | 0.52 (0.34, 0.80) | 0.003** | 0.62 (0.40, 0.95) | 0.030* |
| **Sequence CRT** |  |  |  |  |  |  |  |  |
| Sequential vs Concurrent | 1.64 (1.06, 2.53) | 0.026* | 1.88 (1.13, 3.13) | 0.015* | 1.78 (1.07, 2.96) | 0.026* | 1.68 (1.01, 2.79) | 0.046* |

**Supplementary Table S3** Baseline characteristics and treatment information before and after IPTWin the I-CRT-I and CRT-I groups..

| **Characteristics** | **Before IPTW** | | | | **After IPTW** | | | |
| --- | --- | --- | --- | --- | --- | --- | --- | --- |
|  | **I-CRT-I**  **N=24, (%)** | **CRT-I**  **N=39, (%)** | **P** | **SMD** | **I-CRT-I***  **N=22.6, (%)** | **CRT-I***  **N=38.2, (%)** | **P** | **SMD** |
| **Sex** |  |  | 0.416 | 0.231 |  |  | 0.945 | 0.012 |
| Male | 21 (87.5) | 31 (79.5) |  |  | 19.2 (84.9) | 32.7 (85.6) |  |  |
| Female | 3 (12.5) | 8 (20.5) |  |  | 3.4 (15.1) | 5.5 (14.4) |  |  |
| **Age** |  |  | 0.139 | 0.139 |  |  | 0.987 | 0.009 |
| <65 | 17 (70.8) | 30 (76.9) |  |  | 16.8 (74.3) | 28.3 (74.2) |  |  |
| ≥65 | 7 (29.2) | 9 (23.1) |  |  | 5.8 (25.7) | 9.9 (25.8) |  |  |
| **Smoking** |  |  | 0.345 | 0.318 |  |  | 0.933 | 0.012 |
| Yes | 15 (62.5) | 30 (76.9) |  |  | 16.0 (70.8) | 27.5 (71.8) |  |  |
| No | 9 (37.5) | 9 (23.1) |  |  | 6.6 (29.2) | 10.8 (28.2) |  |  |
| **ECOG PS** |  |  | 0.708 | 0.097 |  |  | 0.917 | 0.013 |
| 0 | 11 (45.8) | 16 (41.0) |  |  | 9.0 (39.8) | 14.7 (38.5) |  |  |
| 1 | 13 (54.2) | 23 (59.0) |  |  | 13.6 (60.2) | 23.5 (61.5) |  |  |
| 2 | 0 (0.0) | 0 (0.0) |  |  | 0 (0.0) | 0 (0.0) |  |  |
| **History** |  |  | 0.927 | 0.089 |  |  | 0.993 | 0.006 |
| Squamous | 19 (79.2) | 32 (82.1) |  |  | 18.9 (83.6) | 31.6 (82.7) |  |  |
| Adenocarcinoma | 4 (16.7) | 6 (15.4) |  |  | 3.0 (13.3) | 5.5 (14.4) |  |  |
| Others | 1 (4.2) | 1 (26) |  |  | 0.7 (3.1) | 1.2 (3.1) |  |  |
| **Stage** |  |  | 0.488 | 0.176 |  |  | 0.918 | 0.017 |
| IIIA | 9 (37.5) | 18 (46.2) |  |  | 9.5 (42.0) | 16.9 (44.2) |  |  |
| IIIB | 10 (41.7) | 17 (43.6) |  |  | 9.8 (43.4) | 17.1 (44.8) |  |  |
| IIIC | 5 (20.8) | 4 (10.3) |  |  | 3.3 (14.6) | 4.2 (11.0) |  |  |
| **Radiation dose** |  |  | 0.120 | 0.387 |  |  | 0.819 | 0.061 |
| <60Gy | 6 (25.0) | 4 (10.3) |  |  | 3.8 (16.8) | 5.6 (14.7) |  |  |
| ≥60Gy | 18 (75.0) | 35 (89.2) |  |  | 18.8 (83.2) | 32.7 (85.3) |  |  |
| **Consolidation ICIS** |  |  | NA | 0 |  |  |  |  |
| Yes | 24 (100.0) | 39 (100.0) |  |  |  |  |  |  |
| No | 0 (0.0) | 0 (0.0) |  |  |  |  |  |  |
| **Sequence CRT** |  |  | 0.434 | 0.201 |  |  | 0.979 | 0.009 |
| Sequential | 17 (70.8) | 31 (79.4) |  |  | 17.4 (77.0) | 29.6 (77.5) |  |  |
| Concurrent | 7 (29.2) | 8 (20.6) |  |  | 5.2 (23.0) | 8.6 (22.8) |  |  |

*Given that IPTW generates weighted counts rather than actual patient numbers, and considering the relatively small sample size, IPTW-adjusted values are presented with one decimal place.

**Supplementary Figure S1** Overall Survival (OS) and Progression-Free Survival (PFS) in the I-CRT and CRT groups before IPTW.

**Supplementary Figure S2** Overall Survival (OS) and Progression-Free Survival (PFS) in the I-CRT and CRT groups after IPTW.
